# Prognostic significance and model evaluation of the modified advanced lung cancer inflammation index in locally advanced resectable gastric cancer patients with neoadjuvant chemotherapy

**DOI:** 10.3389/fonc.2026.1821468

**Published:** 2026-06-04

**Authors:** Weidong Xie, Yaoyu Song, Zhaojie Yu, Huanhao Zhang, Shaoliang Han

**Affiliations:** 1 Department of Head and Neck Surgery, Affiliated Hospital of Jiaxing University (The First Hospital of Jiaxing), Jiaxing, China; 2 The First School of Medicine, School of Information and Engineering, Wenzhou Medical University, Wenzhou, China; 3 School of Public Health & Management, Wenzhou Medical University, Wenzhou, China; 4 Department of Gastrointestinal Surgery, The First Affiliated Hospital of Wenzhou Medical University, Wenzhou, China

**Keywords:** gastric cancer, modified advanced lung cancer inflammation index (mALI), neoadjuvant chemotherapy (NAC), predictive model, prognosis

## Abstract

**Background:**

Sarcopenia is closely associated with poor outcomes in cancer patients. However, its prognostic significance in advanced gastric cancer (GC) patients undergoing neoadjuvant chemotherapy (NAC) remains unclear. This study investigated the prognostic value of the modified Advanced Lung Cancer Inflammation Index (mALI) in this population.

**Methods:**

We retrospectively included 209 patients with locally advanced resectable GC undergoing NAC followed by curative-intent surgery. Preoperative mALI after NAC was assessed using X-tile-derived cutoffs and sex-specific quartiles. OS was analyzed using Cox regression and Bayesian sensitivity analyses, and a postoperative nomogram was internally evaluated using C-index, calibration, DCA, and bootstrap resampling.

**Results:**

Patients were divided into high-mALI group and low-mALI group according to the mALI values, with cut-off values of 10.5 for males and 12.3 for females. Among 209 patients, high mALI was independently associated with improved OS (HR=0.52, 95% CI: 0.30-0.89, P=0.017). Bayesian sensitivity analyses yielded consistent results, and quartile-based analysis showed a protective trend with higher mALI (P for trend = 0.033). The mALI-based nomogram showed acceptable internal performance, with an apparent C-index of 0.765, an optimism-corrected C-index of 0.750, generally acceptable calibration, and potential clinical usefulness on DCA. Adding mALI to a base model including chemotherapy regimen, ypT stage, and ypN stage improved model fit (likelihood ratio test, P=0.012).

**Conclusions:**

Preoperative mALI after NAC was associated with OS in patients with locally advanced resectable GC undergoing NAC followed by surgery. The mALI-based nomogram may provide supplementary postoperative prognostic information but requires external validation before clinical implementation.

## 1 Introduction

Gastric cancer (GC) remains one of the leading malignant tumors that cause cancer-related mortality, ranking fourth among all malignant tumors in terms of death rates (1). Sarcopenia is a progressive and systemic skeletal muscle disorder often associated with adverse outcomes such as falls and mortality (2). Previous studies have found a close association between sarcopenia and the prognosis of cancer patients (3, 4).

Currently, several studies on the relationship between sarcopenia and gastric cancer are mainly focused on patients who have not undergone preoperative neoadjuvant chemotherapy (NAC) (5–7). However, few studies have investigated the prognosis of sarcopenia in advanced gastric cancer patients undergoing NAC followed by surgery (8). Moreover, the impact of NAC on patients may be multifaceted. Because chemotherapy drugs have been found not only to directly cause skeletal muscle loss (9), but also to induce chronic systemic inflammation (10), which is currently considered an important factor contributing to sarcopenia (11). In addition, the level of individual inflammation has been found to be closely related to the prognosis of cancer patients (12).

A recent multicenter study involving over ten thousand patients from China found that the modified advanced lung cancer inflammation index (mALI) can effectively predict prognosis in cancer cachexia patients (13). This index combines sarcopenia-related indicators and inflammatory markers, offering a comprehensive assessment of patients’ nutritional and inflammatory status. However, the prognostic value of mALI in advanced gastric cancer patients undergoing NAC remains unexplored. Therefore, the aim of this study was to investigate the prognostic value of mALI in gastric cancer patients with NAC and to establish and attempt to evaluate relevant predictive models.

## 2 Methods

### 2.1 Study population

The consecutive adults with histopathologically confirmed primary gastric cancer who received neoadjuvant chemotherapy (NAC) with curative intent followed by surgical resection at our the First Affiliated Hospital of Wenzhou Medical University between May 2017 and May 2023, were enrolled in this study. Clinical staging (cTNM) was based on AJCC 8th edition. Eligible patients were defined as those with clinically resectable, locally advanced, M0 (cT2–4 and/or cN+, cM0) on baseline imaging. All cases were reviewed by a multidisciplinary team and were considered candidates for curative (R0) resection prior to NAC. All patients received postoperative adjuvant chemotherapy, and none received postoperative radiotherapy.

The inclusion criteria were as follows: (1) preoperative NAC; (2) subsequent curative-intent gastrectomy at our center; (3) histologically confirmed primary gastric cancer.

Patients were excluded if they met any of the following criteria: (1) distant metastasis (M1) at baseline; (2) other synchronous primary malignancies; (3) massive ascites before surgery; (4) missing essential data required for mALI calculation or subsequent statistical analyses (e.g., preoperative laboratory data within two weeks before surgery, height, or weight); (5) Pre-operative organ failure, active infection, or persistent inflammatory state; (6) unavailable survival outcome data.

The measurement of mALI is defined as occurring after completion of NAC and within two weeks before surgery.

This study was approved by the Ethics Committee of the First Affiliated Hospital of Wenzhou Medical University (KY2024-R253).

### 2.2 Measurement of body composition and definition of mALI

Appendicular skeletal muscle mass (ASM) was measured using Wen et al.’s anthropometric equation specific to the Chinese population, which demonstrated good agreement with dual-energy X-ray absorptiometry (DXA) (14, 15). The equation was:


ASM=0.193×weight(kg)+0.107×height(cm)−4.157×sex(male=1, female=2)−0.037×age(year)−2.631

Appendicular skeletal muscle mass index (ASMI) was calculated after standardizing by height, as follows:


$$ASMI=\frac{\text{A}SM}{\text{height}{\left(\text{m}\right)}^{2}}$$

The mALI formula was defined as follows:


$$mALI=\text{ASMI}\times \frac{\text{Albumin}\left(\text{g}/\text{L}\right)}{\text{NLR}}$$

### 2.3 Data collection and definitions

The following clinical pathological variables were collected: gender, age, BMI, history of diabetes, hypertension, smoking, drinking, abdominal surgery, surgical approach (laparoscopy or laparotomy), chemotherapy regimen, tumor location, pathological classification, and ypT and ypN stage after NAC (AJCC 8th edition). In this study, abdominal surgery history was defined as any prior operation that entered the peritoneal cavity (laparoscopic or open; excluding endoscopic or percutaneous procedures), and surgical approach referred to the surgical approach for the index gastrectomy, recorded on the day of resection.

BMI was defined as weight (kg)/height² (m²). NLR (neutrophil-to-lymphocyte ratio) was defined as neutrophil count (10^9^/L)/lymphocyte count (10^9^/L). SII (systemic immune-inflammation index) was defined as platelet count (10^9^/L) × (neutrophil count (10^9^/L)/lymphocyte count (10^9^/L)). Overall survival (OS; days) was defined as the time from surgery to death due to any cause or the end of follow-up. Follow-up was conducted through a combination of telephone interviews, accessing patient medical records and the regional medical system, ensuring accurate tracking of patient survival status by cross-referencing the results from these methods to minimize loss to follow-up. The follow-up cutoff was May 2024.

### 2.4 Statistical analysis

Continuous variables were described using means (standard deviation), while categorical variables were described using frequencies (percentages). Student’s t-test was used to compare means between low and high mALI groups if continuous variables conformed to normality; otherwise, the Wilcoxon rank sum test was used. For categorical variables, the chi-square test and Fisher’s exact test were used to compare percentages between groups. The optimal cut-off value of mALI was determined using X-tile software (16). A restricted cubic spline (RCS) function was used to evaluate the relationship between mALI and OS in patients undergoing NAC for gastric cancer. The Kaplan-Meier method and log-rank test were used to compare OS between high- and low-mALI patients. Cox proportional hazards regression analyses were conducted to assess independent risk factors affecting OS in gastric cancer patients. Bayesian analysis was conducted using the ‘brms’ package (17) to verify the Cox regression results from a prior probability perspective. To reduce reliance on the X-tile-derived dichotomous cutoff, mALI was also categorized into sex-specific quartiles for supplementary Cox regression analysis. For the Bayesian Cox regression, we specified an informative prior for the mALI based on published data (HR=2.09, 95% CI: 1.842–2.371) (13). Bayesian sensitivity analyses using weakly informative and diffuse priors were further performed, and Markov chain Monte Carlo (MCMC) diagnostics were assessed. In subgroup analyses, the relationship between mALI and OS in different subgroups of NAC patients was evaluated comprehensively. For model establishment, variables with P< 0.05 in univariable Cox regression were entered into the multivariable Cox model, and backward stepwise elimination based on the Akaike information criterion (AIC) was also performed. The variables retained by the two approaches were compared descriptively. For model diagnostics, the proportional hazards assumption was assessed using Schoenfeld residuals for both the full adjusted model and the final nomogram model. Multicollinearity among candidate predictors was examined using generalized variance inflation factors (GVIFs). The time-dependent ROC curves were used to compare the performance of mALI with traditional nutritional and inflammatory markers, as well as to assess the predictive model’s accuracy at different time points. Additionally, C-index, optimism-corrected C-index, calibration slope, calibration curves, and decision curve analysis (DCA) were used to comprehensively evaluate the model. Model performance was further assessed using 1000 bootstrap resamples to evaluate the stability of discrimination, calibration, and decision curve analysis. In this study, P< 0.05 was considered statistically significant. All analyses were performed using R software (version 4.4.3; http://www.r-Project.org).

## 3 Results

### 3.1 Clinical characteristics

Based on the inclusion criteria, a total of 209 patients were included in the study. Patients were divided into high-mALI group and low-mALI group according to the mALI values, with cut-off values of 10.5 for males and 12.3 for females. The clinical characteristics between the high-mALI and low-mALI groups were compared, and the results showed the high mALI patients had the following characteristics such as a higher proportion of males, higher BMI, and a higher proportion of patients with no history of abdominal surgery. The specific distribution between groups was listed in **Table 1**.

**Table 1:** Baseline characteristics of mALI patients.

Variable	N	Overall, N=209^1^	Low, N=84^1^	High, N=125^1^	P-value^2^
Age	209	65 (9)	65 (10)	66 (8)	>0.9
Gender	209				<0.001
Female		57 (27%)	36 (43%)	21 (17%)	
Male		152 (73%)	48 (57%)	104 (83%)	
BMI	209	22.1 (3.2)	21.0 (3.0)	22.8 (3.1)	<0.001
Hypertension	209				0.6
No		129 (62%)	50 (60%)	79 (63%)	
Yes		80 (38%)	34 (40%)	46 (37%)	
Diabetes	209				>0.9
No		172 (82%)	69 (82%)	103 (82%)	
Yes		37 (18%)	15 (18%)	22 (18%)	
Smoke	209				0.080
No smoke		104 (50%)	48 (57%)	56 (45%)	
Smoke		105 (50%)	36 (43%)	69 (55%)	
Alcohol	209				0.2
No alcohol		106 (51%)	47 (56%)	59 (47%)	
Alcohol		103 (49%)	37 (44%)	66 (53%)	
Abdominal surgery	209				0.039
No abdominal surgery		153 (73%)	55 (65%)	98 (78%)	
Abdominal surgery		56 (27%)	29 (35%)	27 (22%)	
Surgical approach	209				0.9
Laparoscopy		138 (66%)	56 (67%)	82 (66%)	
Laparotomy		71 (34%)	28 (33%)	43 (34%)	
Location	209				0.035
Antrum		100 (48%)	36 (43%)	64 (51%)	
Body		54 (26%)	18 (21%)	36 (29%)	
Fundus		53 (25%)	28 (33%)	25 (20%)	
Whole		2 (1.0%)	2 (2.4%)	0 (0%)	
Pathology	209				0.4
Other		5 (2.4%)	3 (3.6%)	2 (1.6%)	
Adenocarcinoma		204 (98%)	81 (96%)	123 (98%)	
NAC	209				0.5
Other		29 (14%)	16 (19%)	13 (10%)	
SOX		150 (72%)	57 (68%)	93 (74%)	
DOS		4 (1.9%)	1 (1.2%)	3 (2.4%)	
FLOT		15 (7.2%)	6 (7.1%)	9 (7.2%)	
XELOX		11 (5.3%)	4 (4.8%)	7 (5.6%)	
ypT	209				0.036
0-2		67 (32%)	20 (24%)	47 (38%)	
3-4		142 (68%)	64 (76%)	78 (62%)	
ypN	209				0.035
0-1		130 (62%)	45 (54%)	85 (68%)	
2-3		79 (38%)	39 (46%)	40 (32%)	

^1^ Mean (SD); n (%).

^2^ Wilcoxon rank sum test; Pearson's Chi-squared test; Fisher's exact test.

BMI, Body mass index; NAC, Neoadjuvant chemotherapy; Chemotherapy regimens: SOX (S-1 and oxaliplatin); DOS (docetaxel, oxaliplatin and S-1); FLOT (5-fluorouracil, leucovorin, oxaliplatin, and docetaxel); XELOX (capecitabine and oxaliplatin).

### 3.2 Prognostic analysis of mALI in patients undergoing NAC for gastric cancer

**Figure 1:**
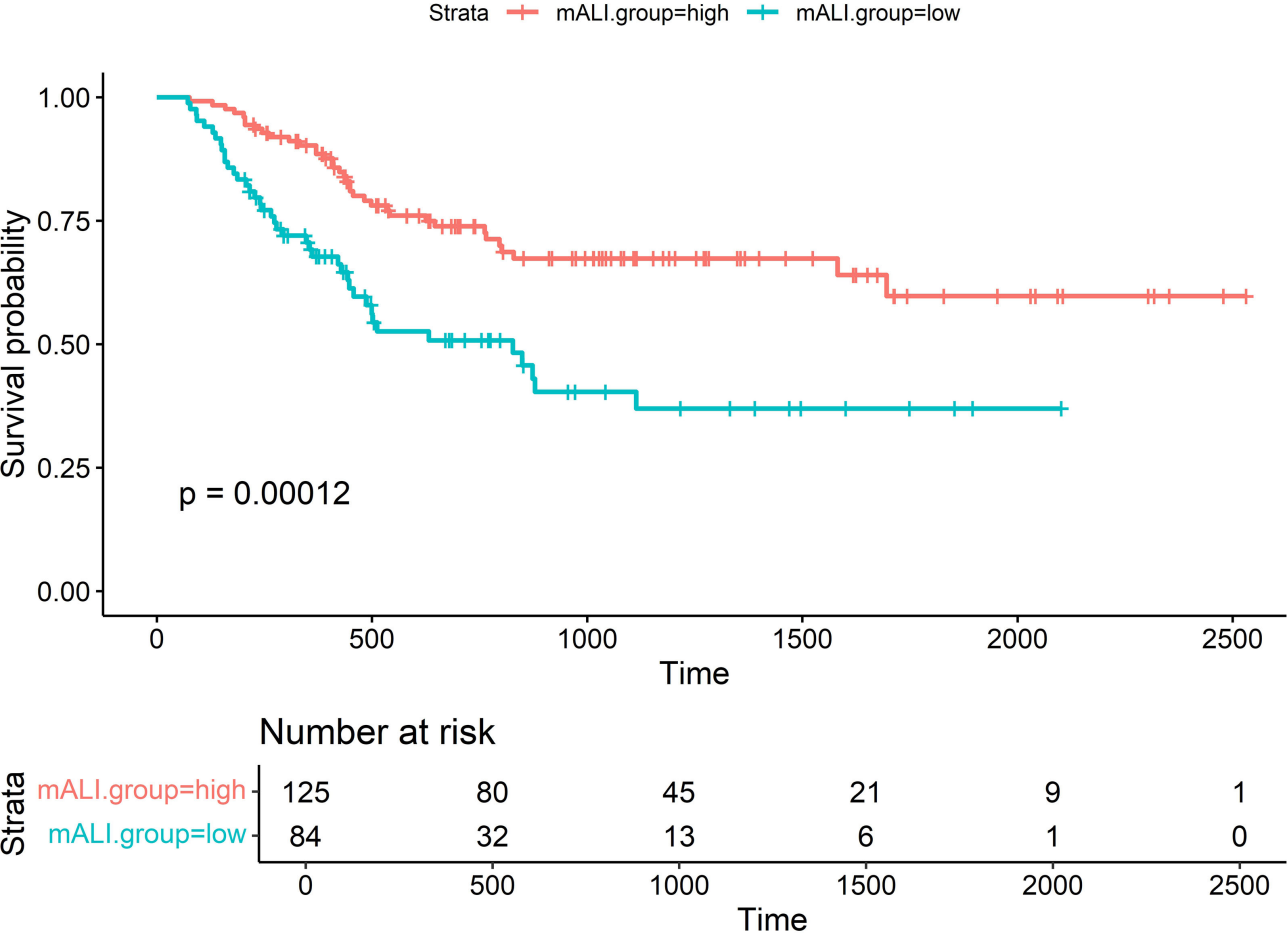
Kaplan-Meier survival curve comparing overall survival between high and low mALI groups.

**Figure 2:**
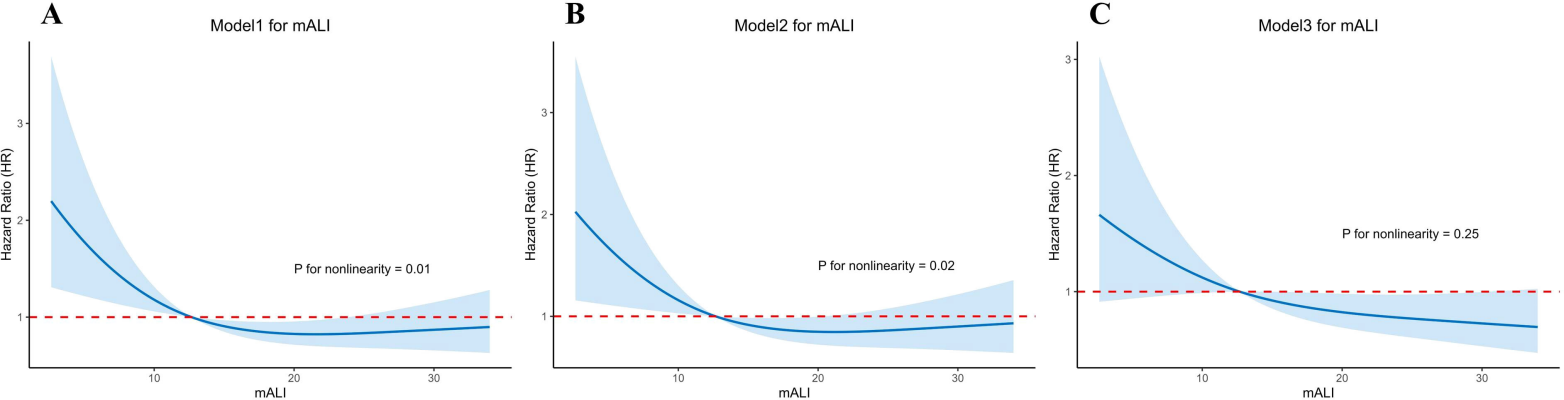
Restricted cubic spline analysis of the association between mALI and hazard ratio for overall survival across three models. **(A)** Model 1, Unadjusted. **(B)** Model 2, Adjusted for gender, age, BMI, hypertension, diabetes, smoking, and alcohol consumption. **(C)** Model 3, Further adjusted for history of abdominal surgery, surgical approach, tumor location, pathology type, chemotherapy regimen, ypT stage, and ypN stage.

**Figure 3:**
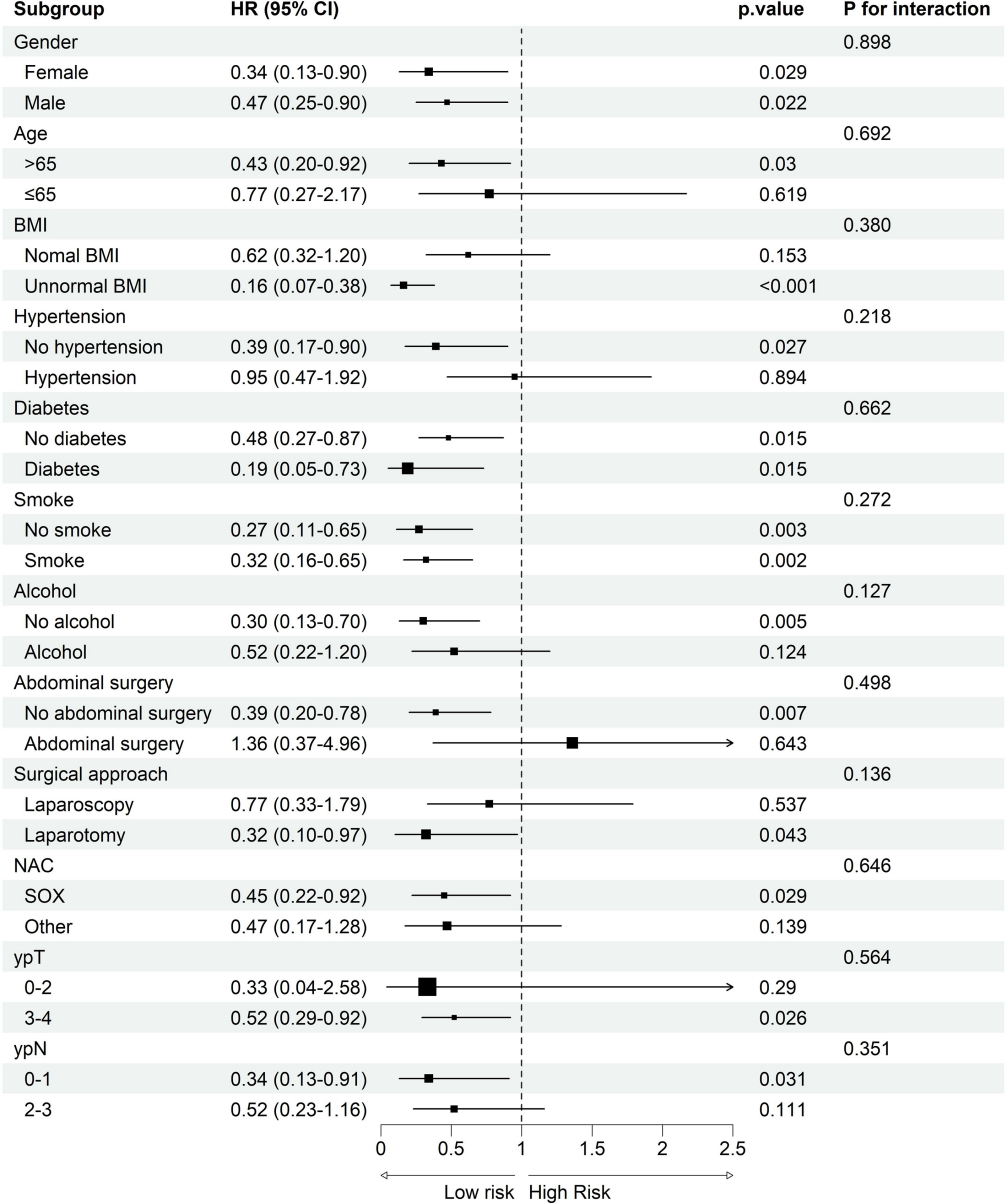
Forest plot of hazard risk of overall survival in various subgroups. The model has been adjusted for gender, age, BMI, hypertension, diabetes, smoke, alcohol, history of abdominal surgery, surgical approach, tumor location, pathology type, chemotherapy regimen, ypT stage, and ypN stage. BMI, Body mass index; ypT, post-neoadjuvant pathological tumor stage; ypN, post-neoadjuvant pathological nodal stage.

In this study, the median follow-up was 974 days, and 132 of 209 patients (63.2%) were censored at the last follow-up. We plotted Kaplan-Meier survival curves and observed that the survival rate of the high-mALI group was significantly higher than that of the low-mALI group (P< 0.001) (**Figure 1**). Subsequently, RCS was used to evaluate whether there was a nonlinear relationship between mALI and OS. The results showed a linear relationship between mALI and OS after multivariable adjustment (**Figure 2**), with insufficient evidence of a non-linear relationship (P = 0.25). Consequently, Cox regression analysis and Bayesian analysis showed that even after adjusting for all covariates, high mALI remained a protective factor for prognosis (Cox: 0.52, P = 0.017; Bayesian: 0.48, posterior probability = 1) (**Table 2**; **Supplementary Material Figure 1**). Bayesian sensitivity analyses using weakly informative and diffuse priors yielded similar results, suggesting that the association between high mALI and improved OS was not driven by the informative prior (**Supplementary Material Tables 1**, **2**). After mALI was categorized into quartiles, higher mALI still showed a protective trend (P for trend = 0.033) (**Supplementary Material Table 3**). In the subgroup analyses, higher mALI generally showed a protective trend across subgroups. However, given the limited sample size and the relatively small number of events in some subgroups, these findings should be interpreted as exploratory (**Figure 3**).

**Table 2:** Cox regression and Bayesian analysis for overall survival.

	Cox regression			Bayesian analysis		
Characteristic	HR^1^	95% CI^1^	P-value	HR^1^	95% CrI^1^	Posterior probability
Model 1						
Low	—	—		—	—	
High	0.42	0.27, 0.67	<0.001	0.47	0.42,0.53	1
Model 2						
Low	—	—		—	—	
High	0.43	0.26, 0.71	<0.001	0.47	0.42, 0.54	1
Model 3						
Low	—	—		—	—	
High	0.52	0.30, 0.89	0.017	0.48	0.43, 0.54	1

Model 1, Unadjusted.

Model 2, Adjusted for gender, age, BMI, hypertension, diabetes, smoking, and alcohol consumption.

Model 3, Further adjusted for history of abdominal surgery, surgical approach, tumor location, pathology type, chemotherapy regimen, ypT stage, and ypN stage.

^1^ HR, Hazard Ratio.

CI, Confidence Interval.

CrI, Credible interval.

### 3.3 Establishment and evaluation of mALI-related model

**Figure 4:**
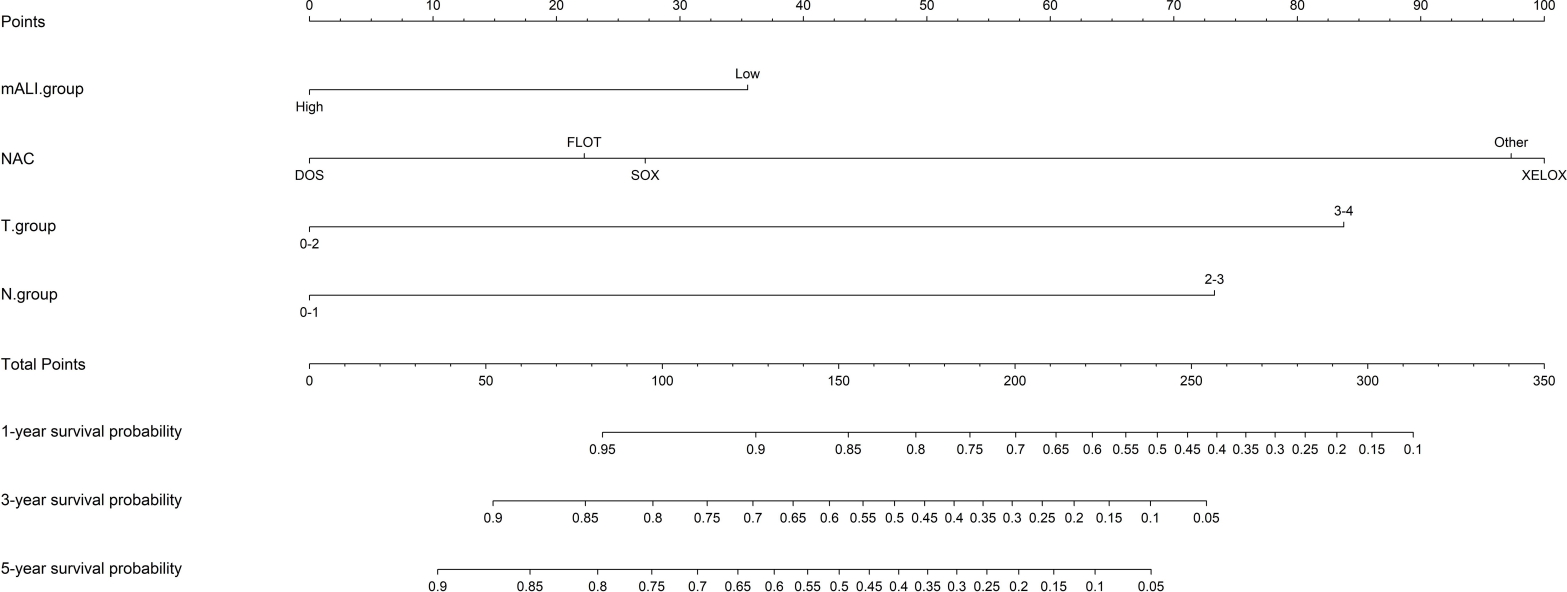
Nomogram for predicting survival in gastric cancer.

On Univariate Cox regression analysis, the overall survival of advanced gastric cancer with NAC was associated with history of abdominal surgery, chemotherapy regimen, ypT stage, ypN stage, and mALI. We then included variables with a univariate analysis P-value of less than 0.05 in the multivariate Cox regression analysis, which indicated that chemotherapy regimen, ypT stage, ypN stage, and mALI were independent prognostic factors for advanced gastric cancer with NAC. Further analysis was conducted using the stepwise backward regression, and the results were consistent with the multivariate Cox regression analysis (**Supplementary Material Table 4**). Model diagnostics showed no apparent violation of the proportional hazards assumption and no substantial multicollinearity among candidate predictors (**Supplementary Material Table 5** and **6**). In addition, we used time-dependent ROC curves to compare mALI with traditional nutritional and inflammatory markers. The results showed that mALI had best predictive power in the first year (AUC=0.716, 95% CI=0.633, 0.798) and better predictive power than most markers in the third and fifth years (**Supplementary Material Figure 2**). A Nomogram was developed based on the factors identified through multivariate Cox regression and stepwise backward regression (**Figure 4**) to predict 1-, 3-, and 5-year OS in advanced gastric cancer patients receiving NAC. The performance of the nomogram was evaluated using 1000 bootstrap resamples, showing an apparent C-index of 0.765 and an optimism-corrected C-index of 0.750; the apparent and optimism-corrected calibration slopes were 1.000 and 0.882, respectively, indicating acceptable discrimination with mild overfitting (**Supplementary Material Table 7**). Compared with the base model including NAC regimen, ypT stage, and ypN stage, the extended model incorporating mALI showed a modest increase in C-index (0.753 to 0.765), a lower AIC (696.88 to 692.60), and improved model fit by likelihood ratio test (χ² = 6.28, P=0.012)(**Supplementary Material Table 8** and **9**). The time-dependent ROC curves for the predictive model showed acceptable performance at 1, 3, and 5 years (**Supplementary Material Figure 3**). The bootstrap-based calibration curves suggested generally acceptable agreement between predicted and observed outcomes, particularly at 1 and 3 years (**Supplementary Material Figure 4**). The DCA curves suggested potential clinical usefulness of the model (**Supplementary Material Figure 5**), with generally consistent patterns observed after bootstrap resampling (**Supplementary Material Figure 6**).

## 4 Discussion

In this study, we further explored the prognostic significance of mALI in advanced gastric cancer patients with NAC based on previous research and established a model to predict overall survival. Our findings indicated that mALI served as an independent prognostic marker for advanced gastric cancer patients with NAC. The model incorporating mALI showed acceptable performance in estimating postoperative survival and may provide supplementary prognostic information for patients with gastric cancer undergoing neoadjuvant chemotherapy.

There is a close relationship between inflammation, cancer, and sarcopenia. In the tumor immune microenvironment, tumors not only recruit inflammatory cells but also utilize these cells, such as tumor-associated macrophages (TAM), to facilitate their own development (18, 19). At the systemic level, elevated inflammation is associated with both the risk of malignant tumor development (20) and tumor progression and adverse prognosis (21). In addition, chronic inflammation is also believed to lead to muscle mass loss. For example, previous studies have shown that interleukin-6 (IL-6) is positively correlated with sarcopenia (22). Cesari et al. (23) further demonstrated that IL-6 and C-reactive protein (CRP) are positively correlated with total fat mass and negatively correlated with appendicular lean mass (ALM). For patients with digestive tract malignancies, especially esophageal and gastric cancers, in addition to the chronic inflammation and tumor-related wasting, patients often experience difficulty in nutrient and energy intake, leading to sarcopenia and malnutrition which may result in poor prognosis (24).

Currently, for potential resectable gastric cancer patients with clinical pathological stage T2N0 and above, it is generally recommended to use neoadjuvant chemotherapy combined with surgical treatment (25). Preoperative chemotherapy is likely to have comprehensive effects on patients. For example, cisplatin, a commonly used chemotherapy drug, is thought to lead to skeletal muscle loss through mechanisms such as myosin loss and inhibition of muscle synthesis (9, 26). Chemotherapy drugs can also activate inflammatory pathways, indirectly causing muscle atrophy (27, 28). Additionally, the subsequent death of cancer cells caused by chemotherapy can also trigger an inflammatory response (29). The introduction of NAC complicates the relationship between inflammation, cancer, and sarcopenia even further. Furthermore, as mentioned earlier, current studies on gastric cancer mainly focus on non-NAC patients, indicating a lack of research in this area. Since Jafri et al. developed the advanced lung cancer inflammation index (ALI) in 2013 (30), ALI has been proven to be useful for predicting prognosis in various cancers (31–34). However, with the increasing prevalence of obesity and the growing attention to sarcopenic obesity, the prognostic value of BMI has been challenged. For cancer patients, muscle mass is not closely related to BMI, even for the same individual, a similar BMI may correspond to different ratios of fat and muscle (35), which can lead to severe muscle loss being overlooked in obese patients. In a study of cancer patients, many obese patients were found to have sarcopenia, which could not be detected by simply observing body weight, as higher weight may mask the presence of sarcopenia (36). The addition of preoperative chemotherapy further complicates the prognostic value of BMI. Considering the key roles that muscle plays in metabolism and immunity (37, 38), we replaced BMI with ASMI in this study, building upon the work of Xie et al. (13), to further explore the prognostic value of mALI in the NAC population with gastric cancer.

Unlike the findings of Xie et al. (13), our study did not find a nonlinear relationship between mALI and OS in gastric cancer patients undergoing NAC; instead, a linear relationship was observed, which may be due to the insufficient sample size. In addition, we noted that the chemotherapy regimen was also included as a parameter in our developed model. Our study showed that the DOS regimen might provide greater benefits to patients compared to other regimens, such as the commonly used SOX regimen. Two neoadjuvant chemotherapy studies on gastric cancer from China, one retrospective study and one phase II clinical trial, both showed that the DOS regimen provided better prognosis compared to the SOX regimen (39, 40). Another retrospective study from China found that the DOS regimen provided greater benefit compared to the XELOX regimen without additional toxicity (41), which is consistent with our results. However, due to the limited sample size, this remains subject to further research.

One of the indices used to calculate the mALI in this study, the ASMI, is derived from common anthropometric characteristics that are easily obtainable in clinical practice. This contrasts with traditional methods such as computed tomography (CT) or dual-energy X-ray absorptiometry (DXA), which not only increase the economic burden on patients but also expose them to ionizing radiation. Bioelectrical impedance analysis (BIA) is relatively more portable and affordable, yet its application can be limited by manufacturer-specific factors and population variations (42). Apart from the readily accessible ASMI, the other indices used to construct the mALI are also easily obtainable. Therefore, the accessibility of mALI may encourage researchers, especially in economically underdeveloped regions, to further explore its clinical significance and potentially provide benefits to patients with gastric cancer.

This study has some limitations. First, it is a single-center retrospective study, lacking multicenter validation, which inevitably introduces some bias. Second, due to the limited sample size, we did not split the original dataset into training and validation sets when building the predictive model. Although we used the Bootstrap method to address this limitation, future large-scale, multicenter prospective studies are needed to externally validate our findings and model performance before clinical implementation. Third, due to practical reasons and the difficulty in obtaining relevant data, we did not investigate the prognostic value of mALI for other outcomes such as recurrence-free survival (RFS). In addition, residual confounding related to treatment heterogeneity cannot be excluded because detailed treatment-related variables were not fully available in this retrospective dataset. Finally, due to the lack of CT- or DXA-based muscle mass measurements, this study could not evaluate the prognostic performance of mALI calculated using imaging- or DXA-derived muscle mass indices.

## 5 Conclusion

In conclusions, preoperative mALI after NAC was associated with overall survival in patients with locally advanced resectable gastric cancer undergoing NAC followed by surgery. The nomogram incorporating mALI, chemotherapy regimen, ypT stage, and ypN stage may provide supplementary postoperative prognostic information. However, this model should be regarded as exploratory and requires external validation in large-scale multicenter prospective studies before clinical implementation.

## Data Availability

The original contributions presented in the study are included in the article/**Supplementary Material**. Further inquiries can be directed to the corresponding author.
