# Supplementary material for: Prognostic significance and model evaluation of the modified advanced lung cancer inflammation index in locally advanced resectable gastric cancer patients with neoadjuvant chemotherapy
Source: Front Oncol. 2026 Jun 4;16:1821468. doi: 10.3389/fonc.2026.1821468 (PMC13275285; doi:10.3389/fonc.2026.1821468)
Supplement: Supplementary file 1 [file DataSheet1.docx]

**Supplementary Material Table 1：Bayesian Sensitivity Analysis for mALI**

| Prior | Posterior HR | HR 95% CrI | Posterior Probability (HR < 1) |
| --- | --- | --- | --- |
| Informative prior | 0.48 | 0.43, 0.54 | 1 |
| Weakly informative prior | 0.53 | 0.32, 0.91 | 0.991 |
| Diffuse prior | 0.51 | 0.30, 0.89 | 0.994 |

Note: HR represents high mALI versus low mALI.

**Supplementary Material Table 2：MCMC Diagnostics for mALI**

| Prior | Parameter | Rhat | Bulk ESS | Tail ESS |
| --- | --- | --- | --- | --- |
| Informative prior | High mALI | 1.00 | 7117 | 2807 |
| Weakly informative prior | High mALI | 1.00 | 4847 | 3549 |
| Diffuse prior | High mALI | 1.00 | 4340 | 3115 |

Note: Rhat close to 1.00 and adequate effective sample sizes indicate satisfactory MCMC convergence.

**Supplementary Material Table 3. Association Between mALI Quartiles and Overall Survival**

| **Model** | **HR** | **95% CI** | **P** |
| --- | --- | --- | --- |
| mALI-Q1 | reference | reference |  |
| mALI-Q2 | 0.99 | 0.51, 1.91 | >0.9 |
| mALI-Q3 | 0.93 | 0.45, 1.90 | 0.8 |
| mALI-Q4 | 0.41 | 0.18, 0.92 | 0.03 |
| *P for trend* |  |  | 0.033 |

Note: All models were adjusted for all covariates.

**Supplementary Material Table 4: Univariable, Multivariable, and Stepwise Cox Regression Analyses**

| **Characteristic** | **Group** | **HR (Univariable)** | **HR (Multivariable)** | **HR (Stepwise backward)** |
| --- | --- | --- | --- | --- |
| **mALI** | Low | Reference | Reference | Reference |
|  | High | 0.42 (0.27-0.67, p<.001) | 0.58 (0.37-0.92, p=.021) | 0.56 (0.35-0.88, p=.012) |
| **Age** | Mean ± SD | 1.00 (0.98-1.03, p=.968) |  |  |
| **Gender** | Female | Reference |  |  |
|  | Male | 0.69 (0.43-1.11, p=.127) |  |  |
| **BMI** | Mean ± SD | 0.97 (0.90-1.04, p=.389) |  |  |
| **Diabetes** | No | Reference |  |  |
|  | Yes | 0.79 (0.44-1.44, p=.450) |  |  |
| **Hypertension** | No | Reference |  |  |
|  | Yes | 1.10 (0.70-1.74, p=.672) |  |  |
| **Smoke** | No smoke | Reference |  |  |
|  | Smoke | 0.84 (0.54-1.32, p=.458) |  |  |
| **Alcohol** | Alcohol | Reference |  |  |
|  | No alcohol | 1.20 (0.77-1.88, p=.425) |  |  |
| **Abdominal surgery** | Abdominal surgery | Reference | Reference |  |
|  | No abdominal surgery | 0.61 (0.38-0.97, p=.036) | 0.72 (0.43-1.19, p=.195) |  |
| **Surgical approach** | Laparoscopy | Reference |  |  |
|  | Laparotomy | 1.51 (0.96-2.36, p=.075) |  |  |
| **Location** | Antrum | Reference |  |  |
|  | Body | 0.82 (0.48-1.42, p=.486) |  |  |
|  | Fundus | 0.75 (0.41-1.35, p=.336) |  |  |
|  | Whole | 3.90 (0.94-16.20, p=.061) |  |  |
| **Pathology** | Other | Reference |  |  |
|  | Adenocarcinoma | 0.48 (0.12-1.95, p=.301) |  |  |
| **NAC** | Other | Reference | Reference | Reference |
|  | SOX | 0.48 (0.27-0.85, p=.012) | 0.34 (0.19-0.63, p=.001) | 0.31 (0.17-0.57, p<.001) |
|  | DOS | 0.48 (0.11-2.08, p=.323) | 0.19 (0.04-0.84, p=.029) | 0.20 (0.04-0.89, p=.035) |
|  | FLOT | 0.44 (0.16-1.21, p=.113) | 0.34 (0.12-0.97, p=.043) | 0.29 (0.10-0.81, p=.018) |
|  | XELOX | 0.69 (0.27-1.78, p=.447) | 1.15 (0.43-3.04, p=.786) | 1.04 (0.40-2.74, p=.930) |
| **ypT** | 0-2 | Reference | Reference | Reference |
|  | 3-4 | 5.96 (2.86-12.42, p<.001) | 3.98 (1.83-8.64, p<.001) | 4.01 (1.84-8.72, p<.001) |
| **ypN** | 0-1 | Reference | Reference | Reference |
|  | 2-3 | 3.95 (2.48-6.29, p<.001) | 3.43 (2.01-5.85, p<.001) | 3.36 (1.97-5.75, p<.001) |

Multivariate candidates: mALI group, history of abdominal surgery, NAC regimen, ypT stage, ypN stage;

Final model (Stepwise backward): mALI group, NAC regimen, ypT stage, ypN stage

**Supplementary Material Table 5: Proportional Hazards Assumption Test Results for the Full and Final Models**

| Model | Variable | Chisq | P |
| --- | --- | --- | --- |
| Full adjusted model | mALI | 0.836 | 0.361 |
| Full adjusted model | Age | 1.919 | 0.166 |
| Full adjusted model | Gender | 0.306 | 0.580 |
| Full adjusted model | BMI | 0.180 | 0.672 |
| Full adjusted model | Diabetes | 0.054 | 0.816 |
| Full adjusted model | Hypertension | 0.469 | 0.493 |
| Full adjusted model | Smoke | 0.511 | 0.475 |
| Full adjusted model | Alcohol | 3.320 | 0.068 |
| Full adjusted model | Abdominal surgery | 0.319 | 0.572 |
| Full adjusted model | Surgical approach | 0.110 | 0.740 |
| Full adjusted model | Location | 0.326 | 0.955 |
| Full adjusted model | Pathology | 0.371 | 0.543 |
| Full adjusted model | NAC | 3.897 | 0.420 |
| Full adjusted model | ypT | 0.591 | 0.442 |
| Full adjusted model | ypN | 0.002 | 0.965 |
| Full adjusted model | GLOBAL | 13.625 | 0.849 |
| Final nomogram model | mALI | 1.134 | 0.287 |
| Final nomogram model | NAC | 3.724 | 0.445 |
| Final nomogram model | ypT | 0.931 | 0.335 |
| Final nomogram model | ypN | 0.136 | 0.712 |
| Final nomogram model | GLOBAL | 5.498 | 0.599 |

**Supplementary Material Table 6. Multicollinearity Assessment Results for the Full and Final Models**

| Model | Variable | GVIF | Adjusted GVIF |
| --- | --- | --- | --- |
| Full adjusted model | mALI | 1.420 | 1.191 |
| Full adjusted model | Age | 1.623 | 1.274 |
| Full adjusted model | Gender | 2.032 | 1.425 |
| Full adjusted model | BMI | 1.453 | 1.205 |
| Full adjusted model | Diabetes | 1.140 | 1.068 |
| Full adjusted model | Hypertension | 1.248 | 1.117 |
| Full adjusted model | Smoke | 2.160 | 1.470 |
| Full adjusted model | Alcohol | 2.257 | 1.502 |
| Full adjusted model | Abdominal surgery | 1.439 | 1.199 |
| Full adjusted model | Surgical approach | 1.202 | 1.096 |
| Full adjusted model | Location | 2.612 | 1.174 |
| Full adjusted model | Pathology | 1.364 | 1.168 |
| Full adjusted model | NAC | 3.347 | 1.163 |
| Full adjusted model | ypT | 1.229 | 1.108 |
| Full adjusted model | ypN | 1.654 | 1.286 |
| Final nomogram model | mALI | 1.039 | 1.019 |
| Final nomogram model | NAC | 1.231 | 1.026 |
| Final nomogram model | ypT | 1.104 | 1.051 |
| Final nomogram model | ypN | 1.327 | 1.152 |

Note: Adjusted GVIF was calculated as GVIF^(1/(2*Df)). Values below 5 indicate no substantial multicollinearity.

**Supplementary Material Table 7**：Internal Validation of the Nomogram Model

| Measure | Apparent | Optimism_corrected |
| --- | --- | --- |
| C-index | 0.765 | 0.750 |
| Calibration slope | 1.000 | 0.882 |

**Supplementary Material Table 8：Model Performance Comparison**

| Model | Variables | C index | AIC | Log likelihood |
| --- | --- | --- | --- | --- |
| Base model | NAC + ypT + ypN | 0.753 | 696.88 | -342.44 |
| Extended model | mALI + NAC + ypT + ypN | 0.765 | 692.60 | -339.30 |

**Supplementary Material Table 9：Likelihood Ratio Test**

| Comparison | Chi square | P value |
| --- | --- | --- |
| Extended model vs base model | 6.28 | 0.012 |

**
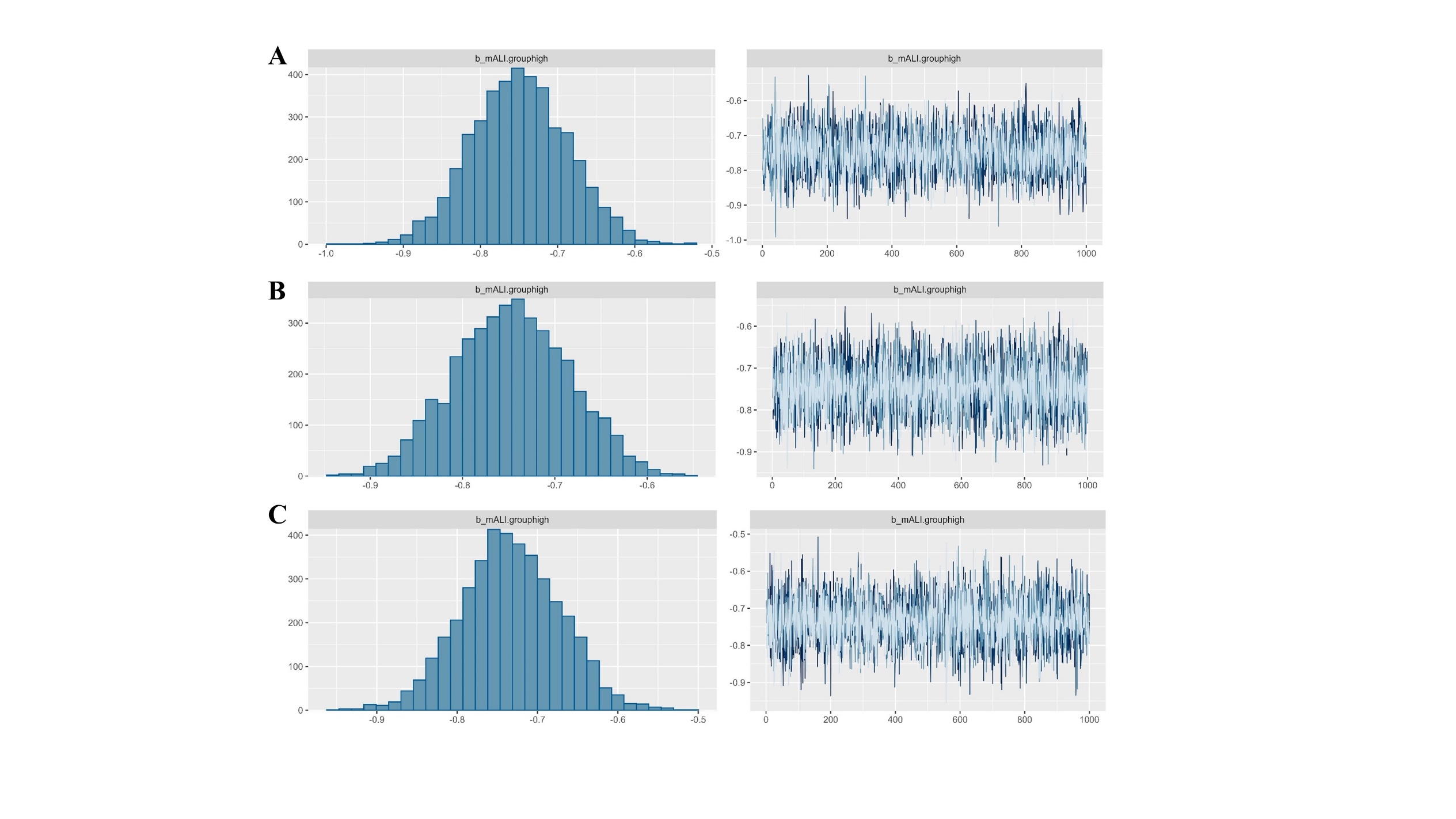
**

**Supplementary Material Figure 1:** **Bayesian Analysis for Overall Survival**

Model 1: Unadjusted.

Model 2: Adjusted for gender, age, BMI, hypertension, diabetes, smoking, and alcohol consumption.

Model 3: Further adjusted for history of abdominal surgery, surgical approach, tumor location, pathology type, chemotherapy regimen, ypT stage, and ypN stage.


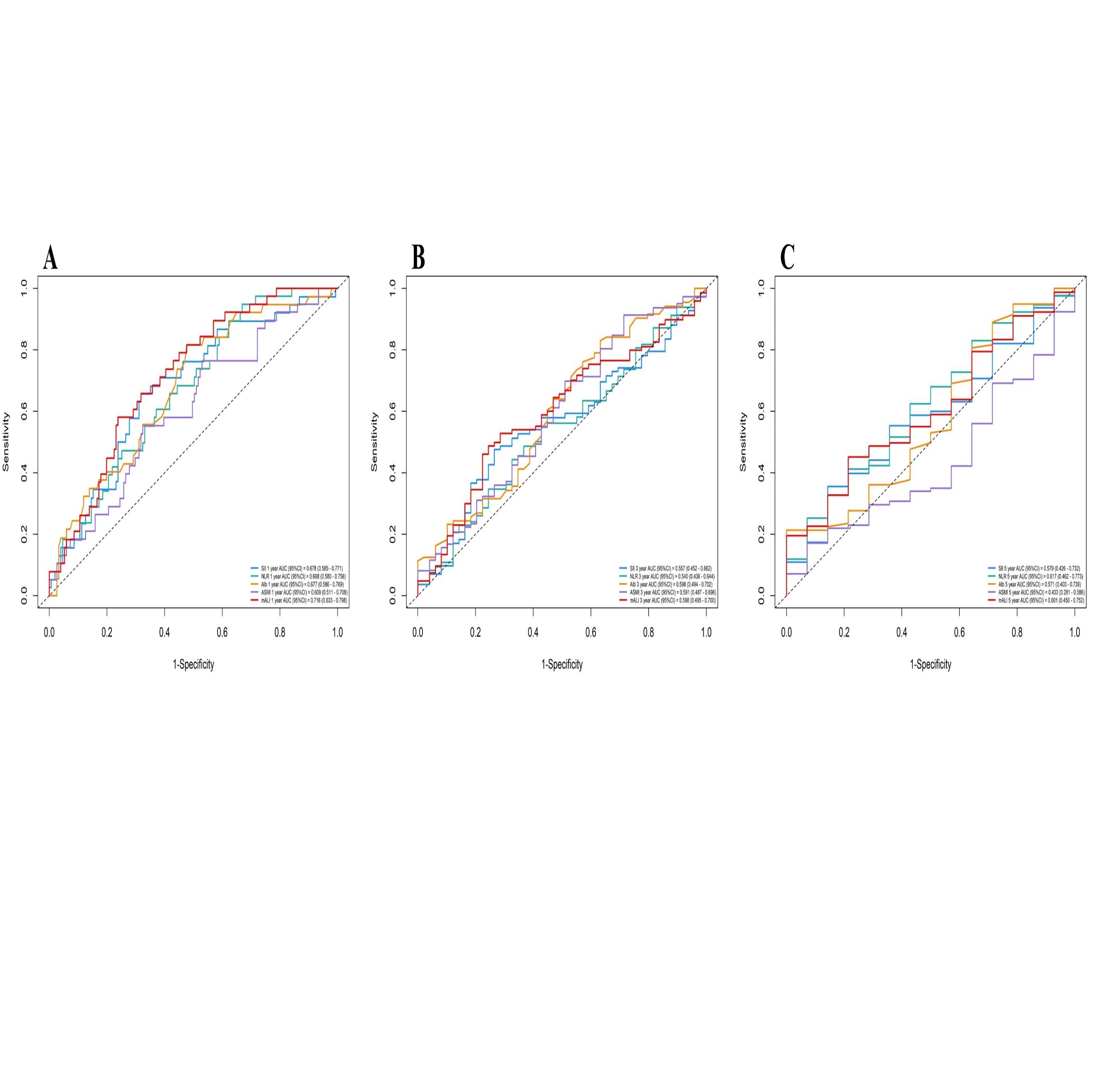


**Supplementary Material Figure 2: Time-dependent ROC Curves for different markers**

A:1-Year; B: 3-Year; C:5-Year


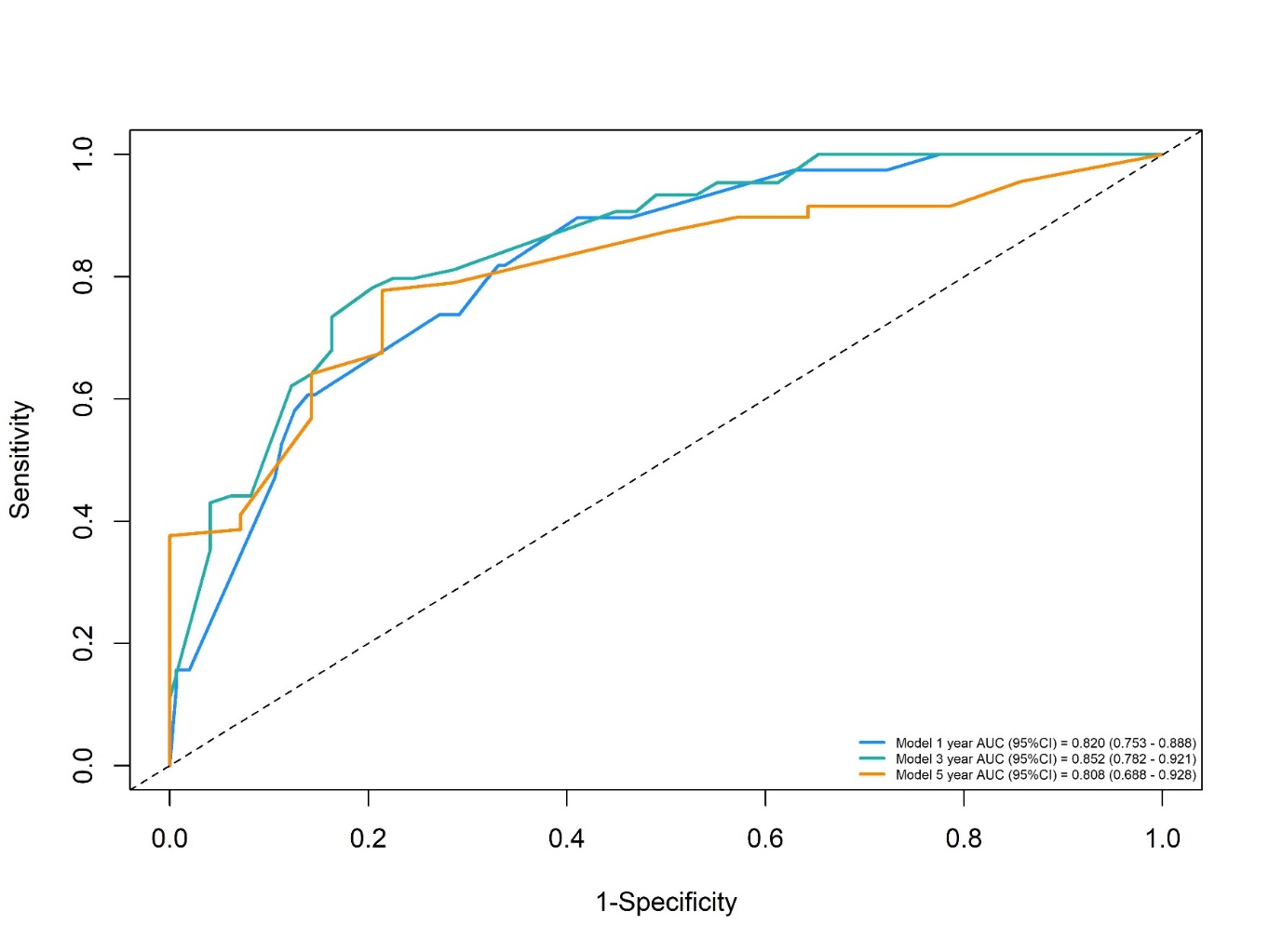


**Supplementary Material Figure 3: Time-dependent ROC Curves for Predictive Model**

**
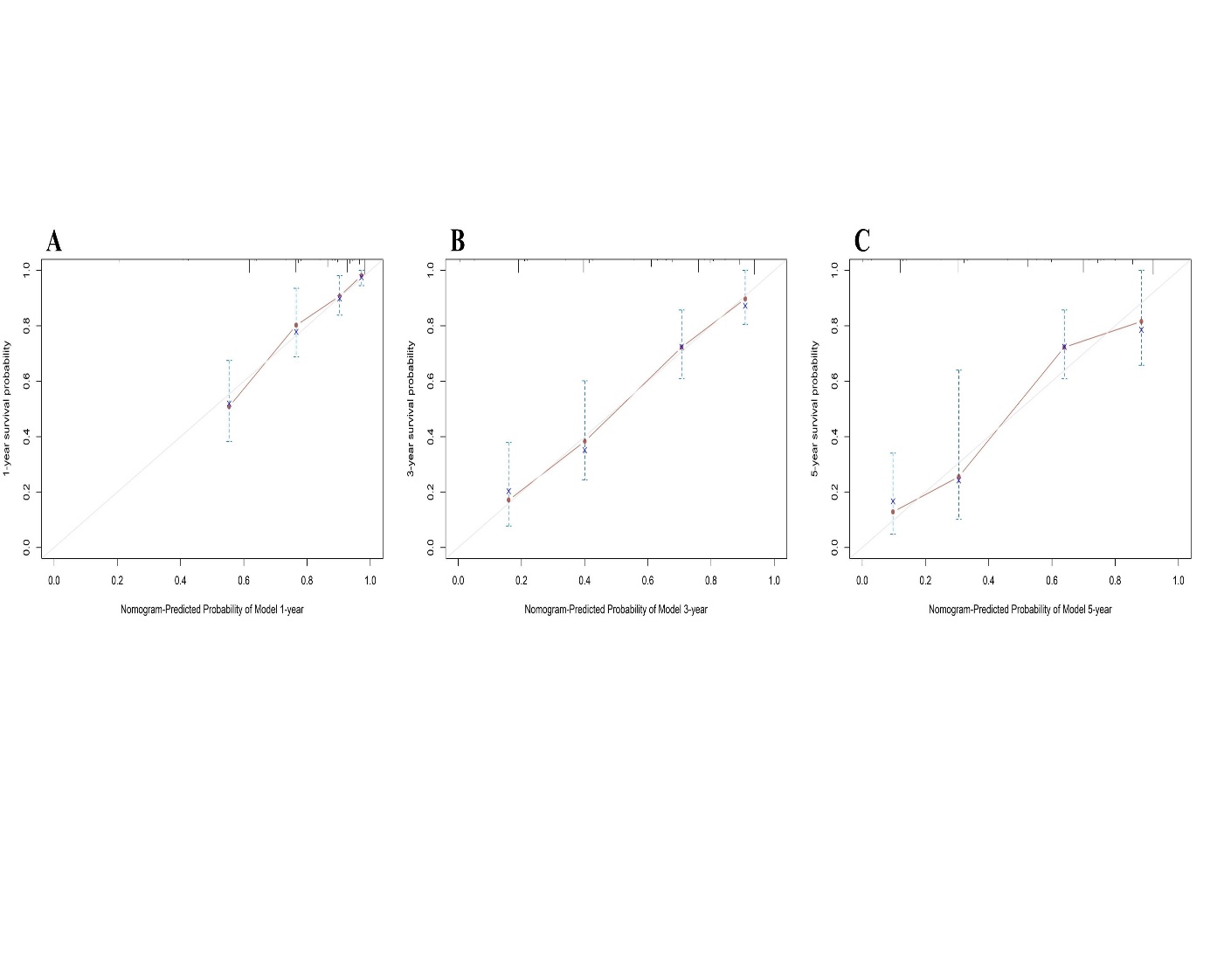
**

**Supplementary Material Figure 4: Calibration Plots for 1-, 3-, and 5-Year Survival Prediction**

A:1-Year; B: 3-Year; C:5-Year


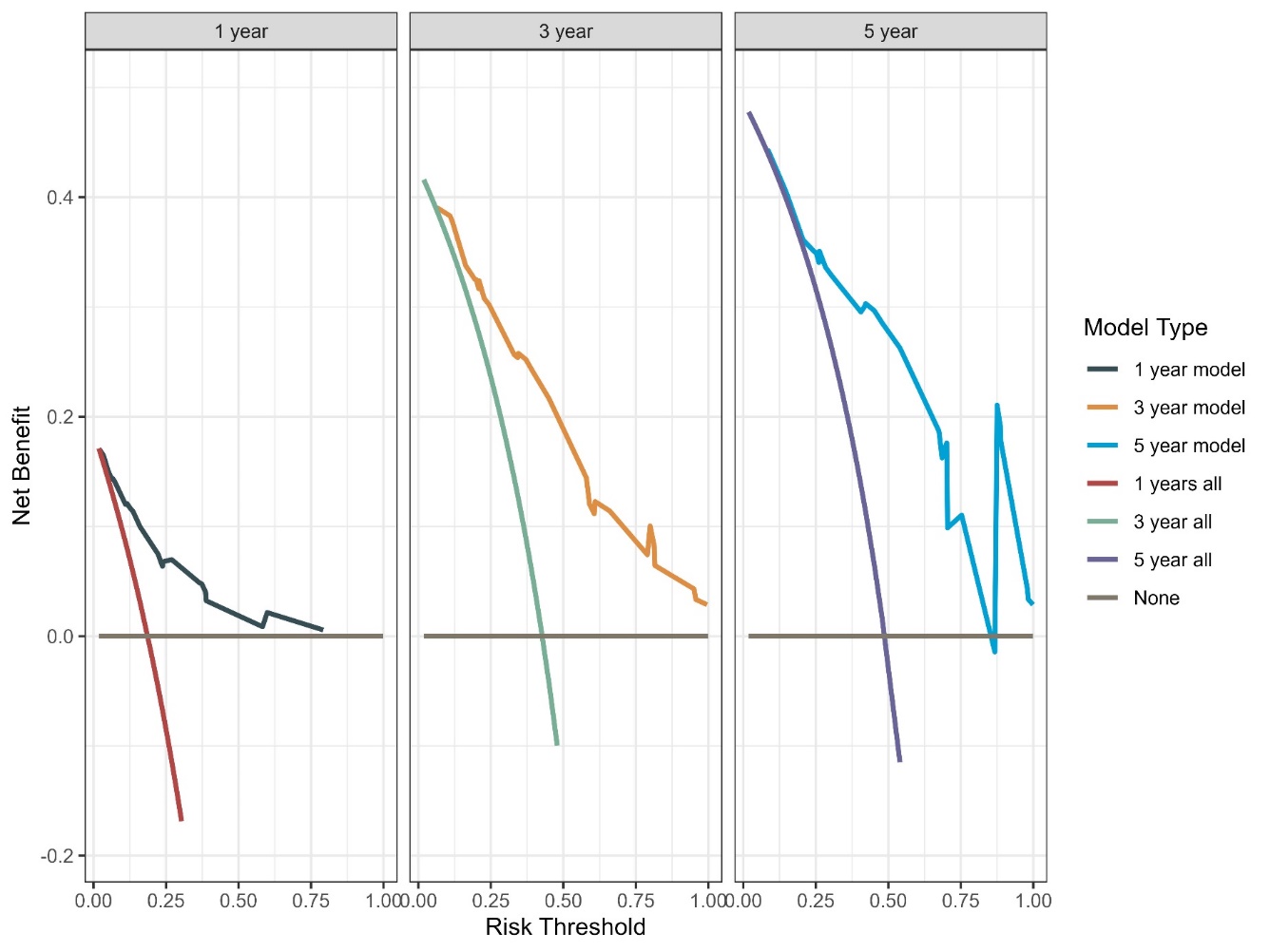


**Supplementary Material Figure 5: DCA Curves for Predictive Model**


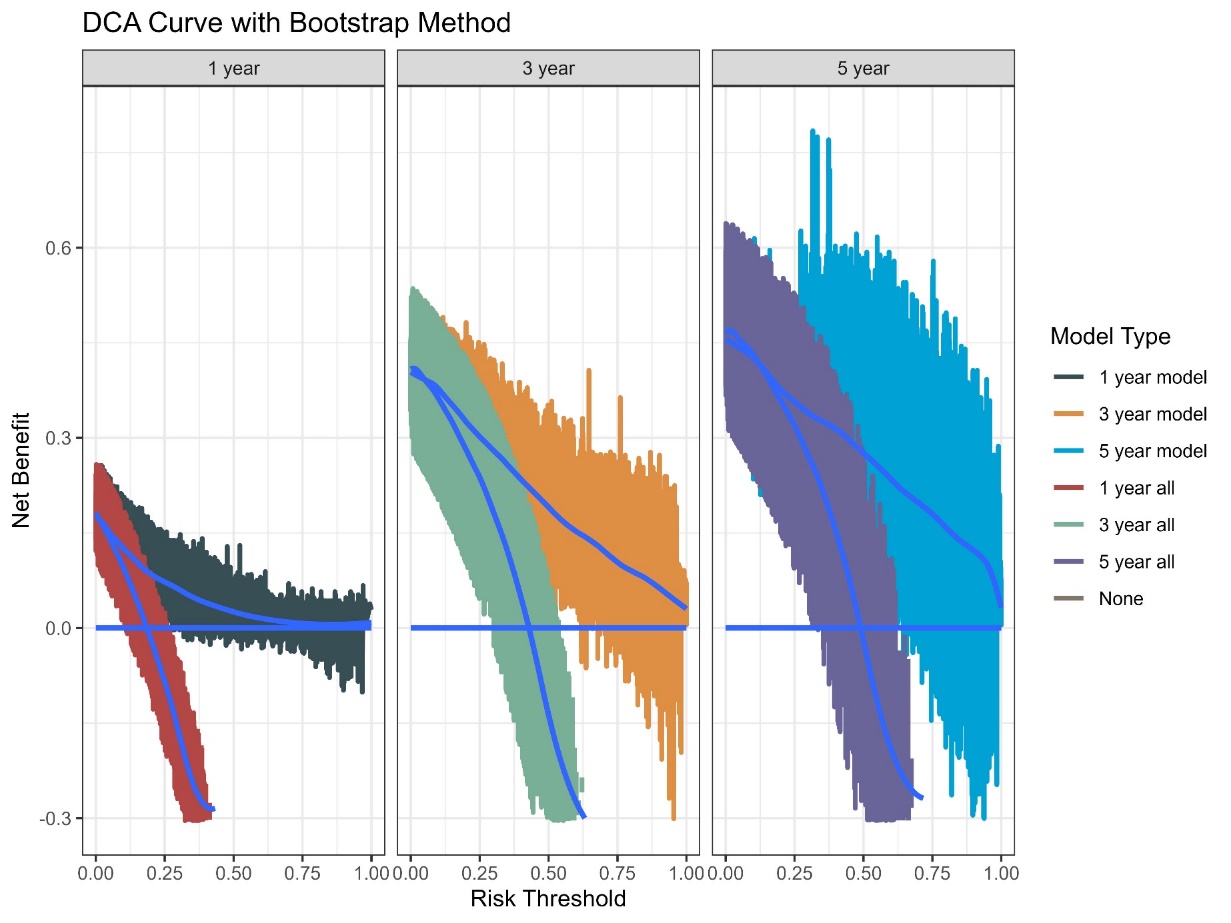


**Supplementary Material Figure 6: DCA Curves with Bootstrap Method for Predictive Model**
